# Identification of hub lncRNAs correlated with tetralogy of fallot based on weighted gene co-expression network analysis

**DOI:** 10.1016/j.bbrep.2024.101786

**Published:** 2024-07-13

**Authors:** Ye Ding, Chun Zhu

**Affiliations:** a Department of Obstetrics and Gynecology, Women's Hospital of Nanjing Medical University, Nanjing Women and Children's Healthcare Hospital, Nanjing, 210004, Jiangsu, China; b Department of Child Health Care, Women's Hospital of Nanjing Medical University, Nanjing Women and Children's Healthcare Hospital, Nanjing, 210004, Jiangsu, China

**Keywords:** Tetralogy of fallot, Weighted gene co-expression network analysis, Hub lncRNAs

## Abstract

The most prevalent cyanotic congenital heart disease, Tetralogy of Fallot (TOF), has an unknown etiology. Long-stranded non-coding RNAs (lncRNAs) have been linked to cardiac development and congenital heart disease, as evidenced by an increasing number of studies; nevertheless, additional research is necessary to fully understand the function that TOF-related lncRNAs play in the condition. This study constructed lncRNA-mRNA co-expression networks, performed functional enrichment analysis, and screened hub lncRNAs using Weighted Gene Co-expression Network Analysis (WGCNA) using the Gene Expression Omnibus dataset GSE36761. Ten hub lncRNAs, including IRF1-AS1, AC012360.6, HLA-F-AS1, RP1-253P7.4, NPTN-IT1, RP11–166P13.4, RP5-1183I21.2, SNHG14, CH17-98J9.1, and RP11–894P9.1, were identified by WGCNA analysis as potentially significant contributors to the development of TOF. Based on functional enrichment analysis, lncRNA mainly contributes to TOF by altering gene splicing patterns. New insights on the mechanism underlying TOF occurrence are provided by identifying hub lncRNAs associated with the disease and analyzing their regulatory networks.

## Highlights

• Weighted gene co-expression network analysis identified hub lncRNAs for TOF.
• A lncRNA-mRNA co-expression network was created.
• Hub lncRNAs may impact mRNA cleavage and cell cycle regulation in patients with TOF.

## 1 Introduction

Tetralogy of Fallot (TOF) is the most common cyanotic congenital heart disease. It includes four types of cardiovascular malformations: right ventricular outflow tract obstruction, misaligned ventricular septal defect, aortic saddle, and secondary right ventricular hypertrophy. The incidence rate for every 1000 live births is 0.34, over 30 % of patients will experience atrial reentrant tachycardia, approximately 10 % of patients will experience severe ventricular arrhythmia and the incidence rate for sudden cardiac death is expected to be 0.2 % annually [1,2]. Following surgery, the majority of patients still experience chronic problems such as pulmonary valve regurgitation, heart failure, and ventricular arrhythmias [3]. Therefore, it is imperative to investigate the pathophysiology and etiology of TOF actively.

Genetic and environmental factors work together to create most congenital heart diseases, including TOF. Over 90 % of them are caused by hereditary or genetic defects, with less than 5 % caused by environmental factors alone [4,5]. A comprehensive understanding of the regulatory mechanism behind cardiac development abnormalities produced by genes highly associated with TOF, such as NK2 homeobox 5 (NKX2-5), GATA binding protein 4 (GATA4), T-box transcription factor 5 (TBX5), etc., remains elusive despite some progress in this area [6]. Therefore, it will be possible to gain fresh insights into the occurrence and development of TOF by actively looking for key regulatory factors. A large and diverse class of RNA molecules longer than 200 nucleotides incapable of encoding proteins is known as long-chain non-coding RNA (lncRNA). They are crucial in regulating heart development, heart function, and heart disease [[7], [8], [9], [10]]. The first lncRNA associated with TOF was lnc HA117, and higher expression of lnc HA117 may be associated with poor prognosis for patients with TOF [11]. Knocking down lncRNA TBX5-AS1:2 causes methylation of the promoter of the crucial transcription factor TBX5, which is involved in cardiac development and contributes to the incidence of TOF by preventing cell proliferation [12]. These findings imply that lncRNA might be very important for the incidence and prognosis of TOF. In the present research on the role of lncRNA in the occurrence and development of TOF, identifying and analyzing essential genes among these identified and reported genes has become a significant concern. Weighted gene co-expression network analysis (WGCNA) provides a quick and accurate solution to this problem. The investigation on using WGCNA for TOF is still in its early stages. This study aimed to investigate further and search for the regulatory network and critical pathways of TOF by incorporating WGCNA and lncRNA-mRNA into the regulatory network.

## 2 Methods

### 2.1 Data sources for weighted gene co-expression network analysis

The sequencing data for this study was sourced from the Gene Expression Omnibus database (https://www.ncbi.nlm.nih.gov/geo/). Transcriptome sequencing data from 22 patients with TOF and four normal human hearts were included in the dataset GSE36761.

### 2.2 Differential gene screening

The R package “DESeq2” data [13] was analyzed in the Gene Expression Omnibus using the classic Bayesian method (Version 3.10.3, http://www.bioconductor.org/packages/2.9/bioc/html/limma.html) provided by the Limma package in R/Bioconductor software (Love, M. I et al., 2014). The screening criteria were adjusted P value < 0.05, |log2FC| >1 for TOF differentially expressed lncRNA and mRNA. Volcano maps of differentially expressed lncRNA and mRNA data exhibit differential expression and adhere to the requirements for presenting sampleexpression data.

### 2.3 WGCNA

To construct a hierarchical clustering tree, screen lncRNAs with a median absolute deviation of the top 75 %. Then, the “PicksoftThreshold” function is used to convert the calculated Pearson correlation matrix into an adjacency matrix. The “Blockwise Modules” function in the WGCNA [14] package can be used to build gene networks and modules. First, we selected an appropriate soft threshold to ensure the constructed network has a power-law distribution. Build a scale-free network by grouping genes with similar expression patterns and identifying gene co-expression modules using the Blockwise modules method. The clustering tree is divided into multiple branches and given various colors for visualization using a dynamic tree-cutting algorithm. Utilize module eigengenes (ME) to summarize each module and determine the significant component that is most essential in each module. This can be done by creating a synthetic gene that represents the expression profile of all genes in a module. The relationship between ME and gene expression levels is known as module membership (MM). Additionally, it determines the gene significance (GS) value of the gene within the module, signifying the association between the gene and the sample. Selecting a soft thresholding power of 10 can improve the adjacency function's ability to meet the scale-free requirement. A total of six distinct gene expression modules were created, with the gray module representing the gene without co-expression and each module having a unique color and set of genes. We analyzed correlation using disease as the primary clinical feature on various gene modules. Based on the MM value, the top 10 high membership lncRNAs were chosen as key lncRNAs in the clinical feature saliency module.

### 2.4 Correlation analysis between lncRNA and mRNA

With P < 0.05 serving as the screening threshold, Metascape was utilized for gene ontology (GO) analysis [15] of differentially expressed mRNA (DEmRNA) to gain a deeper understanding of the gene function in TOF. We conducted GO functional analysis on the identified lncRNA dataset module [16] and examined the lncRNA mRNA correlation coefficient. Choose the top 10 mRNA with the highest correlation coefficient for GO analysis with each important lncRNA. The R package “clusterProfiler” was used to implement GO analysis [17].

## 3 Results

### 3.1 Screening for differentially expressed genes in the public dataset GSE36761

**Fig. 1:**
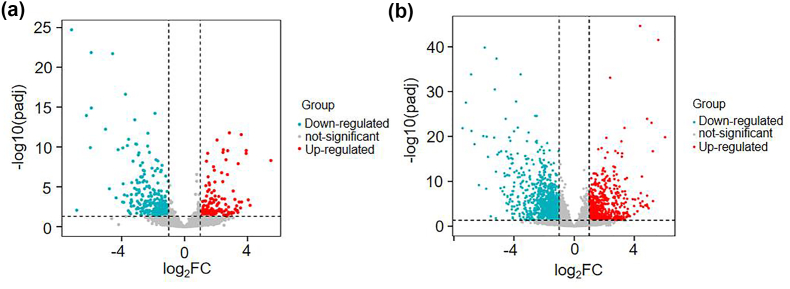
Volcano plot of differential gene expression. A: lncRNA; B: mRNA. The horizontal dashed line indicates the significance horizontal line, and the vertical dashed line represents the difference multiple line.

Using the common dataset GSE36761, lncRNAs and mRNAs linked with TOF were analyzed in this study. Transcriptome sequencing data from 22 patients with TOF and four healthy human hearts are included in this dataset. Following quality control and data normalization, 418 lncRNAs that were differentially expressed compared to standard samples were found; 137 were upregulated, and 281 were downregulated (Fig. 1A). A total of 1880 differentially expressed mRNA were discovered by differential expression analysis (padj <0.05, |log2FC| >1), of which 709 were upregulated and 1171 were downregulated (Fig. 1B). Red dots in the figure represent genes that are significantly upregulated by differences, whereas genes that are downregulated considerably by differences are represented by blue dots.

### 3.2 Construction of weighted gene co-expression network and screening of key modules

**Fig. 2:**
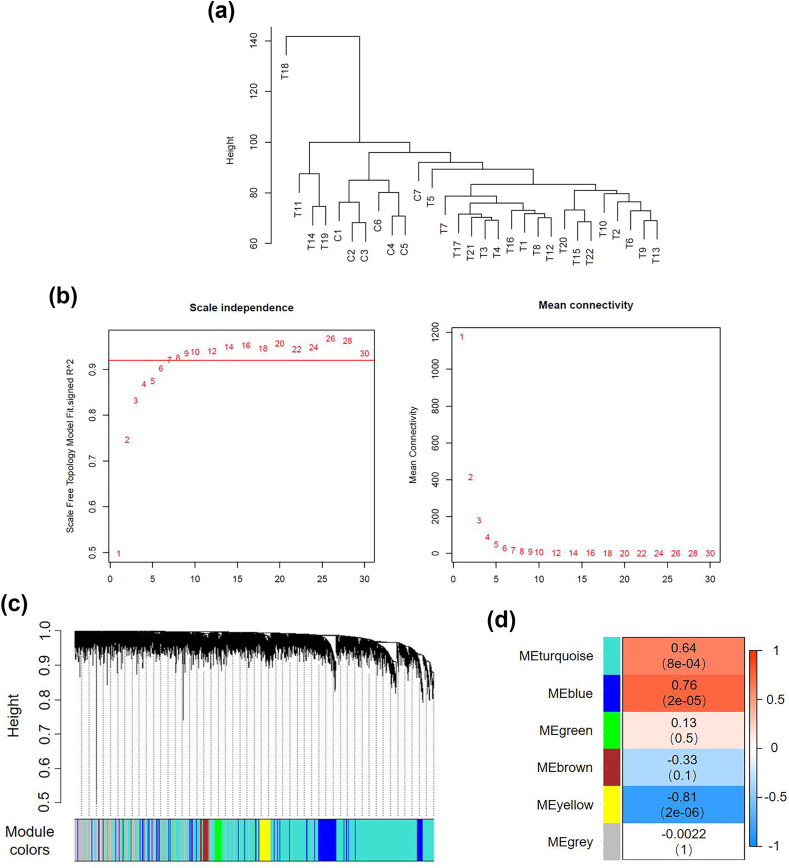
Construction of weighted gene co expression network and screening of key modules. A: Sample hierarchical clustering to detect outliers; B: Analysis of network topology for soft thresholding parameters; C: Gene Cluster Tree and Gene Module; D: Correlation heatmap between clinical features and module features.

We performed weighted gene co-expression network analysis on lncRNA to better understand its function in TOF. To create a hierarchical clustering tree, the first 75 % of the median absolute deviation of lncRNAs was screened (Fig. 2A). Samples C7, T11, T14, T18, and T19 had notable outliers. The residual samples were kept to build a weighted co-expression network. This study's adjacency function better satisfied the scale-free requirement when the ideal soft threshold of 10 was chosen (Fig. 2B). As a result, the network is closer to the actual state of the biological network and has a power law distribution.

Fig. 2C displays the resulting gene clustering tree diagram and each module's colors. Six distinct gene expression modules were built; the gray module represents the gene without co-expression. Determine the correlation between every module's features and calculations separately. The typical gene is identified as the first principal component in the principal component analysis of each co-expression module. Characteristic gene expression patterns from several co-expression modules are associated with clinical characteristics. We used disease as the primary clinical feature and performed correlation analysis using disease gene modules. MEblue had a 0.76 correlation coefficient and was substantially correlated with disease (P = 2e−05) (Fig. 2D). MEblue was chosen as the primary module as a result.

### 3.3 Screening of key lncRNAs related to TOF

**Fig. 3:**
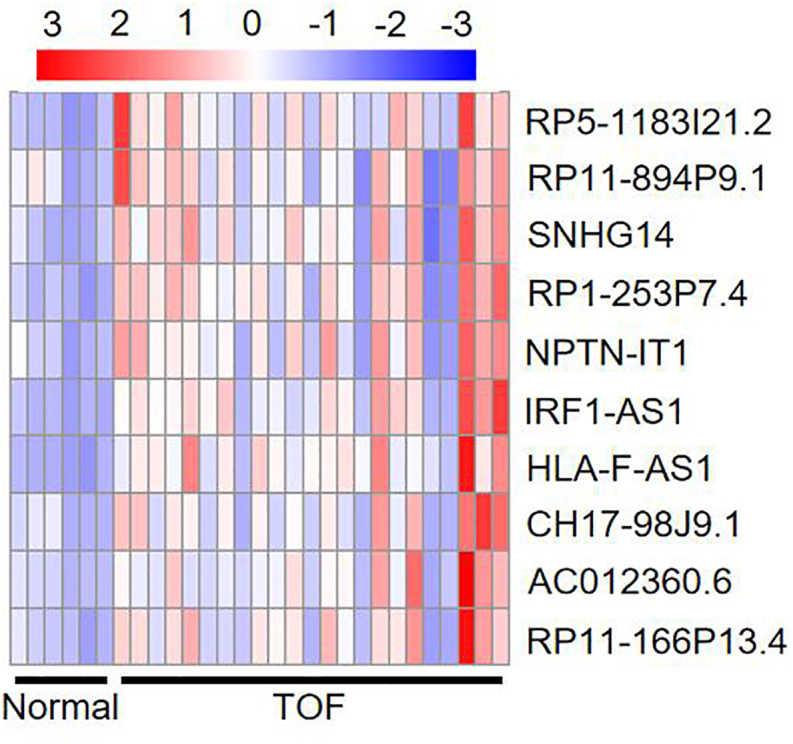
Heat map of key lncRNAs. The greater the intensity of red color, higher is the relative abundance of the genus, and the greater the intensity of blue color, lower is the relative abundance of the genus.

The top 10 high membership lncRNAs were identified as key lncRNAs in the clinically significant modules (Table 1) based on the MM value. These modules include IRF1-AS1, AC012360.6, HLA-F-AS1, RP1-253P7.4, NPTN-IT1, RP11–166P13.4, RP5-1183I21.2, SNHG14, CH17-98J9.1, and RP11–894P9.1. Compared to the normal control group, the expression levels of these 10 lncRNAs were significantly greater in patients with TOF (Fig. 3, with red denoting high expression and blue denoting low expression).

**Table 1:** 10 key lncRNAs.

Gene name	MM value
IRF1-AS1	0.926573404
AC012360.6	0.917025702
HLA-F-AS1	0.91496465
RP1-253P7.4	0.913276875
NPTN-IT1	0.911506787
RP11–166P13.4	0.911476119
RP5-1183121.2	0.908067717
SNHG14	0.904174974
CH17-98J9.1	0.903332913
RP11–894P9.1	0.902981016

### 3.4 Construction of lncRNA and mRNA co-expression network

**Fig. 4:**
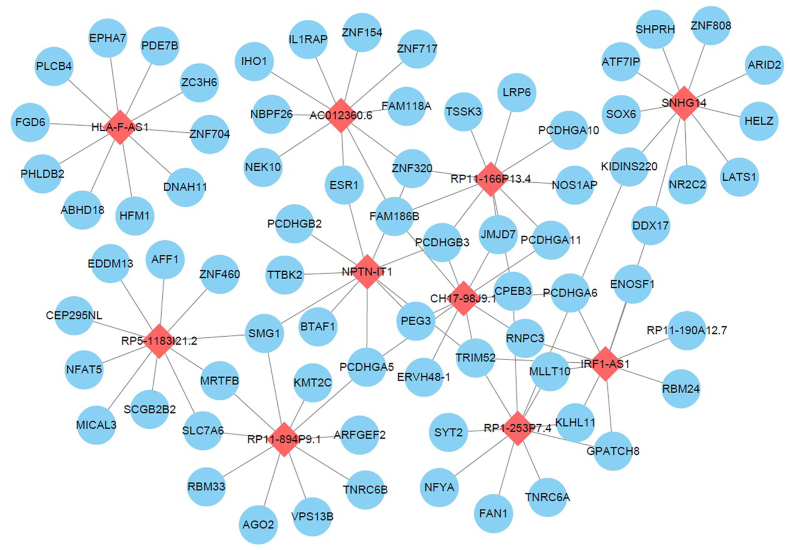
LncRNA and mRNA co-expression network. The red diamonds represent lncRNAs while the blue circles represent mRNAs.

Based on earlier study findings, 1880 differential mRNAs and 418 differential lncRNAs were discovered, which may have a role in controlling the onset and progression of TOF. Based on the regulatory interactions between mRNA and lncRNA, paired genes/lncRNA expression profiles from disease-related co-expression modules were integrated into the gene expression dataset to identify the interaction network of lncRNA-mRNA in TOF. This allowed researchers to investigate the functions of the ten significant lncRNAs mentioned above. A lncRNA-mRNA co-expression network was created by examining the Pearson correlation coefficient of lncRNA-mRNA in the clinically significant module (Fig. 4).

**Fig. 5:**
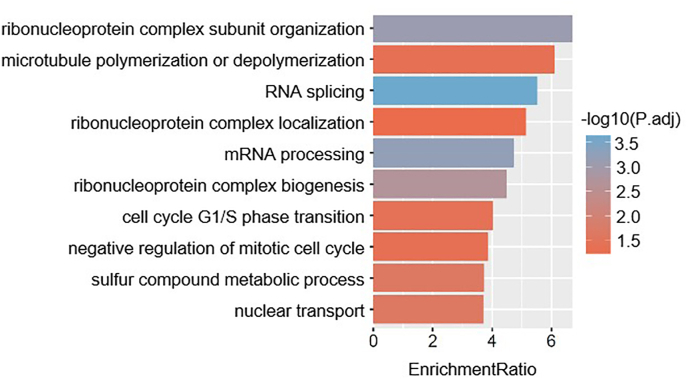
GO enrichment analysis of co-expressed genes. The top 10 GO enrichment terms of the top 10 mRNA with the highest correlation coefficient with each critical lncRNA.

To further investigate the pathophysiology of TOF, we chose the top 10 mRNA with the highest correlation coefficient, with each critical lncRNA as the node of the co-expression network. We then indirectly provided feedback on the biological function of crucial lncRNAs through GO enrichment analysis of these genes. The ribonucleoprotein complex subunit organization, microtubule aggregation or disaggregation, RNA splicing, localization of ribonucleoprotein complex, mRNA processing, ribonucleoprotein complex biogenesis, G1/S phase transition of cell cycle, negative regulation of mitotic cell cycle, sulfur compound metabolism process, and nuclear transport are the top 10 GO enrichment analysis results (Fig. 5), among others. These findings imply that important lncRNAs may impact mRNA cleavage and cell cycle regulation in the cardiac tissue of patients with TOF in the clinically critical module.

## 4 Discussion and conclusions

WGCNA, or joint expression network analysis, is popular for demonstrating the correlation between genes and disease types or clinical phenotypes. It is used to find strongly associated gene clusters in gene expression data. Consequently, essential genes within biologically connected modules might be identified by WGCNA as prospective biomarkers and therapeutic targets for future research [14]. Luo et al. [18] identified 1977 co-expressed target genes for the important lncRNA MFI2-AS in the study of new clinical indicators for advanced colon cancer. According to enrichment analysis of putative target genes, the prognosis and course of colon cancer are primarily influenced by these genes through interactions with the cell cycle and cytokine receptors. After cardiopulmonary bypass surgery, Liu et al. [19] discovered using WGCNA analysis that IL-6 may mediate myocardial fibrosis in children with TOF. WGCNA analysis was utilized in this study to pinpoint important TOF-related lncRNAs, namely SNHG14 and HLA-F-AS1. The majority of current research on these lncRNAs focuses on cancer. For instance, lncRNA SNHG14 can function as a molecular sponge to absorb various miRNAs and contribute to developing breast, colorectal, and pancreatic cancer [[20], [21], [22]]. Furthermore, it has been observed that patients with ischemic stroke exhibit aberrant expression of lncRNA SNHG14, which is thought to simulate cell apoptosis, angiogenesis, inflammation, and cell death [23]. Additionally, it can function as a sponge to adsorb miR-322–5p and miR-384–5p, upregulating the expression of protocadherin 17 (PCDH17) and aggravating myocardial hypertrophy response [24]. The only information available suggests that lncRNA HLA-F-AS1 can promote colorectal cancer cell proliferation, migration, and invasion, inhibit cell apoptosis, and accelerate the advancement of colorectal cancer [25,26]. By focusing on the miR-21–3p/PEG3 axis, which is anticipated to emerge as a new therapeutic target, lncRNA HLA-F-AS1 can also prevent the growth of ovarian cancer [27]. It has, however, sporadically been linked to cardiovascular diseases. This work discovered HLA-F-AS1 differential expression in TOF for the first time; more investigation is required to determine its role in the onset and progression of TOF.

Nowadays, lncRNA function studies are typically conducted by inferring the lncRNA function from target gene functions that are known to exist [28,29]. The lncRNA mRNA co-expression network is frequently used to predict target genes. It is possible to identify shear regulatory factors like RNA binding motif protein 24 (RBM24) among the co-expressed genes. RBM24 is a member of the RNA-binding protein family and is expressed selectively in the heart and skeletal muscle. It is also highly conserved. It is one of the biomarkers of early myocardial differentiation and is crucial for sarcomere development and cardiac contraction. Targeted inactivation of RBM24 has been shown in studies [30] to interfere with cardiac development and sarcomere formation in striated muscle. The rationale is that RBM24, the primary regulatory factor for the selective splicing of striated muscles, regulates many muscle-specific splicing events. These splicing events are essential for the growth and pathology of myocardial and skeletal muscles. Subsequent research has revealed that the endocardial cushion, a derived structure of the heart membrane and valves, may grow abnormally if RBM24 is absent. Congestive heart failure may result from abnormalities in the endocardial cushion [31]. This is in line with the GO enrichment analysis findings of this study, which indicates that lncRNAs associated with TOF might be involved in regulating mRNA splicing, potentially impacting physiological processes like heart development.

It was widely accepted that cell enlargement is how the heart develops after birth. Nonetheless, research indicates that the number of myocardial cells in the human left ventricle multiplies by 3.4 times between the ages of one and 20, with neonates having the highest percentage of myocardial cells undergoing mitosis and cytoplasmic division [32]. Myocardial cell cycle and cytokinesis activity were significantly reduced in patients with TOF and pulmonary artery stenosis (TOF/PS) [33], suggesting that myocardial cells maintain normal cell cycle activity and may be essential for normal development of the heart from birth to adolescence. The GO enrichment analysis results of this study suggest that the regulation of cell transformation from the G1 phase to the S phase and mitosis may be mediated by the essential lncRNAs in the clinically significant modules. Numerous studies have discovered that lncRNA can control the expression of downstream target genes at multiple levels, which controls the expression of cell cycle regulatory factors and influences the cell cycle process [34]. Moreover, lncRNA can also change the expression of different cell cycle regulatory factors. However, the role and mechanism of lncRNA linked to TOF in myocardial cell cycle control have not been documented in the literature and warrant additional investigation. Currently, such study is primarily focused on tumor cells.

## Funding

The author(s) received no financial support for the research, authorship, and/or publication of this article.

## CRediT authorship contribution statement

**Ye Ding:** Writing – original draft. **Chun Zhu:** Project administration.

## Declaration of competing interest

The authors declare that they have no known competing financial interests or personal relationships that could have appeared to influence the work reported in this paper.
